# Ileocaecal Intussusception with a Lead Point: Unusual MDCT Findings of Active Crohn's Disease Involving the Appendix

**DOI:** 10.1155/2015/856483

**Published:** 2015-10-19

**Authors:** Ebru Ozan, Gokce Kaan Atac, Egemen Akincioglu, Mete Keskin, Kamil Gulpinar

**Affiliations:** ^1^Department of Radiology, Ufuk University School of Medicine, 06520 Ankara, Turkey; ^2^Department of Pathology, Ufuk University School of Medicine, 06520 Ankara, Turkey; ^3^Department of General Surgery, Ufuk University School of Medicine, 06520 Ankara, Turkey

## Abstract

Adult intussusception is a rare entity accounting for 1% of all bowel obstructions. Unlike intussusceptions in children, which are idiopathic in 90% of cases, adult intussusceptions have an identifiable cause (lead point) in the majority of cases. Crohn's disease (CD) may affect any part of the gastrointestinal tract, including the appendix. It was shown to be a predisposing factor for intussusception. Here, we report a rare case of adult intussusception with a lead point, emphasizing diagnostic input of multidetector computed tomography (MDCT) in a patient with active CD that involves the appendix.

## 1. Introduction

Crohn's disease (CD) is a chronic granulomatous inflammatory disease of the gastrointestinal tract, which can involve almost any segment from the mouth to the anus [1]. CD can involve the appendix extending from the terminal ileum or the cecum. Appendiceal involvement is seen in about 25% of patients with ileal CD [2].

Intussusception is the invagination of a bowel loop with its mesenteric fold (intussusceptum) into the lumen of a contiguous portion of bowel (intussuscipiens) as a result of peristalsis. A few cases of small bowel or colonic intussusceptions in patients with Crohn's disease have been reported; hence CD is considered as a predisposing factor for intussusception [3]. In adults, intestinal obstruction due to intussusception is a relatively rare phenomenon and it accounts for minority of intestinal obstructions [4].

Different imaging techniques have been proposed for the diagnosis of intussusception. Although ultrasound is the modality of choice in children, computed tomography (CT) is mandatory in adults to make the diagnosis, to determine the underlying cause, to find the lead point, and to evaluate the complications [5].

We describe an unusual case of acute intestinal obstruction due to ileocaecal intussusception in a patient with CD. The case also presents multidetector CT (MDCT) findings of active CD with appendiceal involvement. To our knowledge, this report is the first that illustrates MDCT findings of intussusception with a lead point and appendiceal involvement in an adult patient with active CD.

## 2. Case Presentation

A 25-year-old male, known to have CD for 5 years, presented to the emergency room with abdominal pain and vomiting. He had no history of previous surgery. A moderate amount of free fluid in the peritoneal cavity with distended bowel loops was described on ultrasound in another institution a few hours before. Physical examination revealed a tenderness in the lower quadrants. Bowel sounds were hypoactive and the rectum showed no feces on digital rectal examination. Routine laboratory investigation of blood sample showed an elevated white blood cell count (15,200/mL).

**Figure 1:**
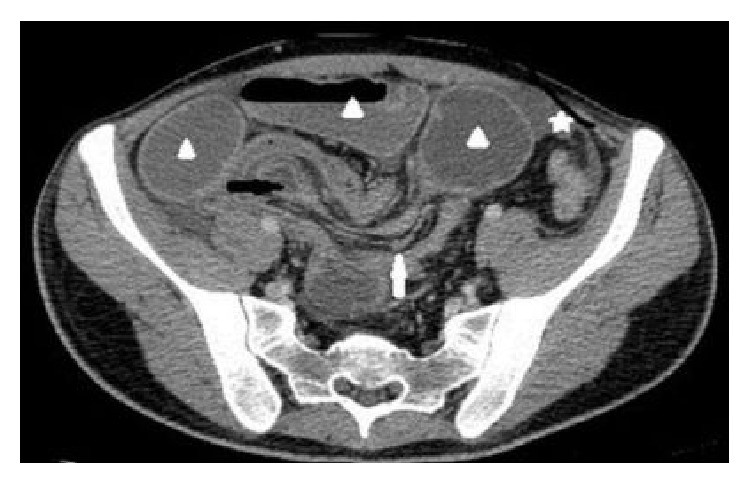
Contrast enhanced axial MDCT image demonstrates the presence of intussusception (black arrow) with an accompanying complex of mesenteric fat and blood vessels (white arrow). Dilated small bowel loops due to obstruction (arrowheads) and a moderate amount of free fluid (five-pointed star) are seen.

**Figure 2:**
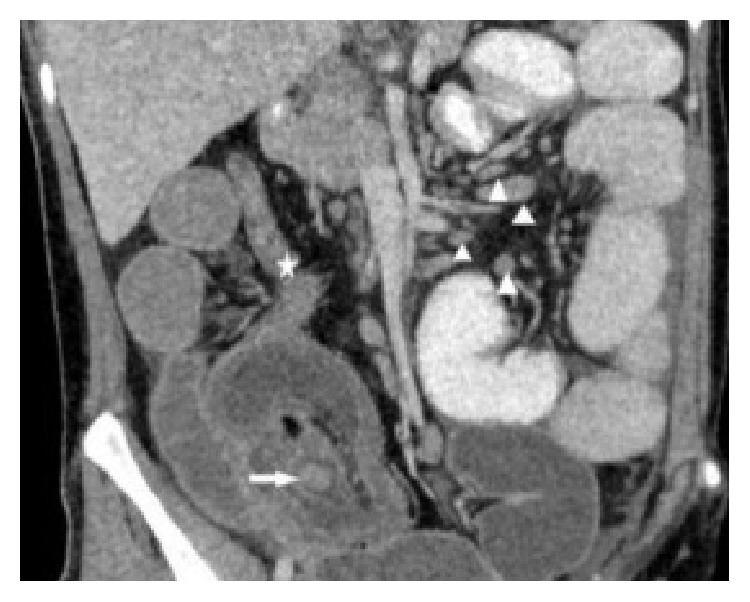
Coronal multiplanar reformatted (MPR) image demonstrates the entire intussusception with a round soft-tissue mass serving as a lead point (white arrow), collapsed colon distal to the intussusception (five-pointed star), and multiple enlarged lymph nodes (arrowheads).

**Figure 3:**
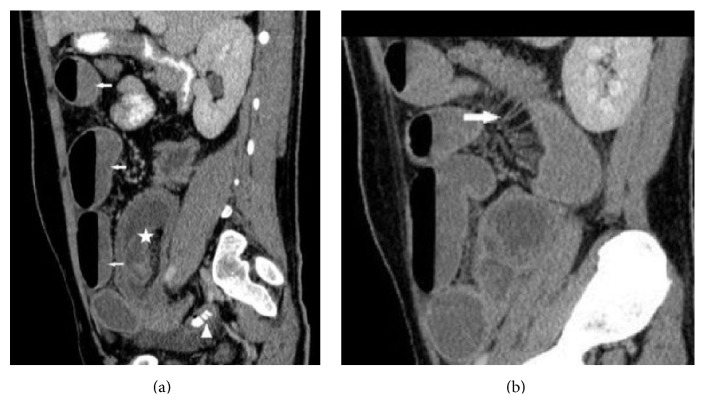
(a) Sagittal MPR images show intussusception (five-pointed star) and dilated loops (arrows). Enlarged appendix showing wall enhancement (arrowhead) is seen. (b) Engorgement of vasa recta, “comb” sign, is nicely demonstrated (arrow).

MDCT of the abdomen with oral (1.5 liters of water mixed with 50 mL sodium and meglumine diatrizoate, Urografin 50 mL, Schering) and IV (ioversol, Optiray 300/100, Mallinckrodt) nonionic iodinated contrast material administration revealed small bowel obstruction due to long segment ileocolic intussusception with invaginated mesenteric fat and vessels, dilated small bowel loops, and moderate amount of free peritoneal fluid (Figure 1). Coronal multiplanar reformatted (MPR) images clearly demonstrated the lead point. Collapsed colonic segment distal to the intussusception and multiple enlarged lymph nodes were also seen (Figure 2). Sagittal MPR images showed enlarged appendix with enhancing wall indicating appendiceal involvement in this patient with CD. “Comb” sign representing engorgement of vasa recta in the mesentery was also nicely demonstrated on sagittal MPR images (Figure 3).

Preoperative diagnosis of ileocaecal intussusception with a lead point was made based on MDCT findings, and also enlarged appendix with wall enhancement was found to be highly suspicious for appendiceal involvement in this case of active CD.

**Figure 4:**
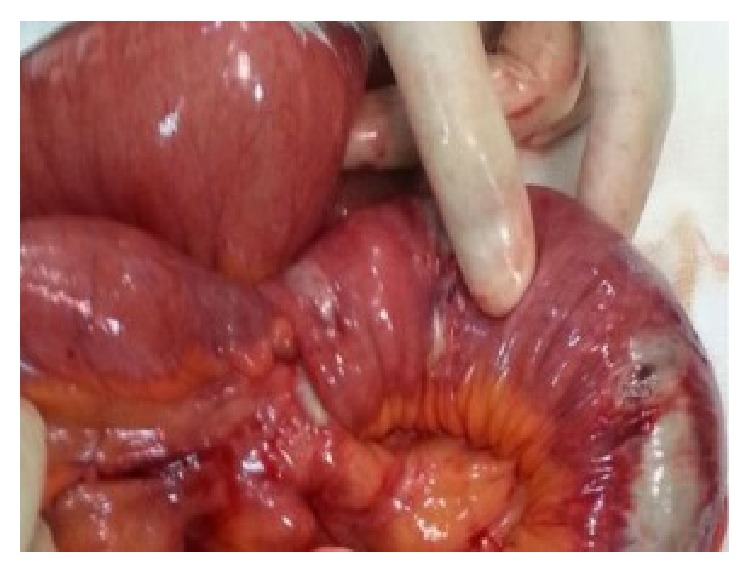
Intraoperative view of the intussusception.

An open surgical laparotomy showed a long segment of ileocaecal intussusception (Figure 4). Appendix was enlarged and inflamed as preoperatively illustrated on MDCT. Surgical resection of the affected bowel segments including terminal ileum, ileocaecal valve, and appendix was performed.

**Figure 5:**
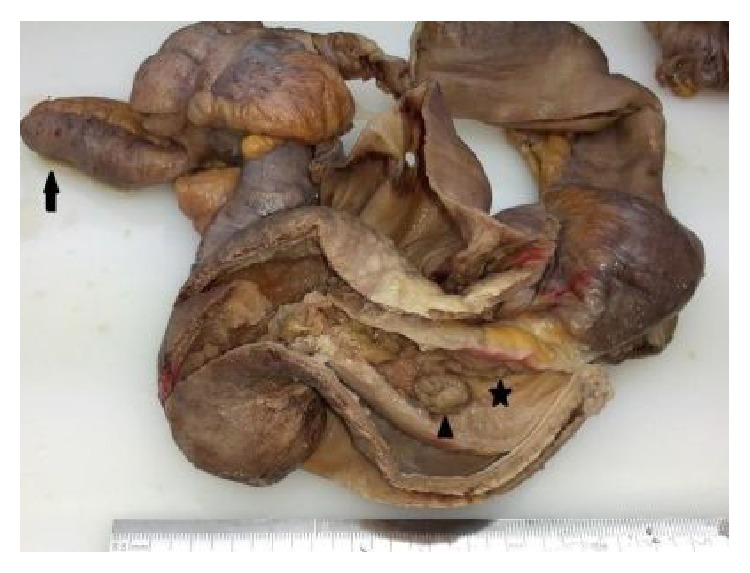
Gross appearance of the resected material composing the invaginated terminal ileum (five-pointed star), polypoid lesion (arrowhead), and enlarged appendix (arrow).

On gross examination the 25 cm long specimen composing terminal ileum, ileocaecal valve, and appendix was evaluated in the pathology department. Terminal ileum was invaginated through the ileocaecal valve and appendix was enlarged. A polypoid lesion, measuring 1,5 cm in diameter corresponding to the lead point, was on the mucosal surface of the invaginated terminal ileum (Figure 5).

**Figure 6:**
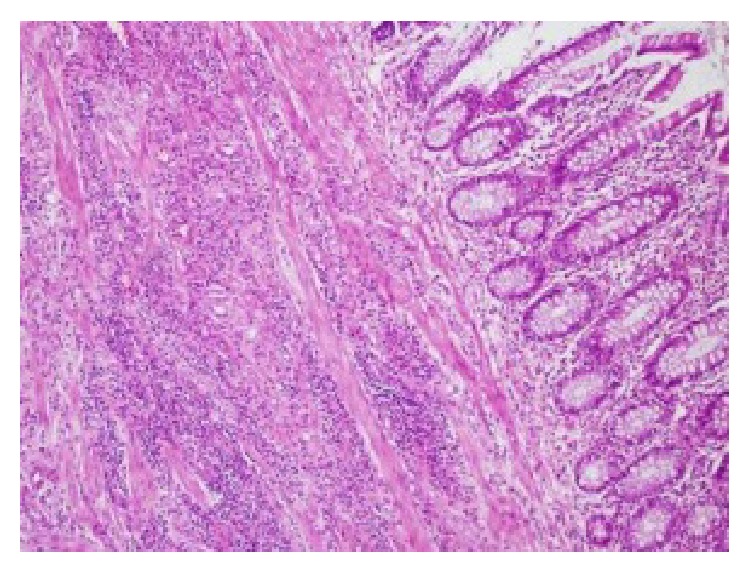
Microscopic examination demonstrates lymphoid aggregates and chronic inflammatory cells on the thick appendiceal wall, H&E stain, ×100.

Microscopic examination revealed that the polypoid lesion (lead point) was an inflammatory polyp. Mucosal glands and lamina propria of the intussusceptum and the intussuscipiens had ischemic changes. There were lymphoid follicles, vasodilatation, and edema in the mucosa of the colonic wall covering the invaginated part. The wall of the appendix was thick (Figure 6). There were lymphoid aggregates and chronic inflammatory cells on the whole section of the appendix and periappendiceal fat tissue. A final diagnosis of inflammatory Crohn's disease affecting the terminal ileum, ileocaecal valve, and appendix was made.

## 3. Discussion

Intussusception occurs when a proximal segment of bowel and its associated mesentery invaginate into the lumen of the adjacent distal segment. It is a rare entity in adults and it accounts for only 1% of all cases of intestinal obstructions and 5% of all intussusceptions [6].

Cases of intestinal obstruction secondary to small bowel intussusception by a segment of active Crohn's ileitis have been rarely reported. There have been reports of intussusception in adult patients with Crohn's disease and history of previous surgery [7]. Transient nonobstructive intussusception in two patients with Crohn's disease was also described and repeated abdominal CT showed spontaneous resolution of intussusceptions in both patients [8]. In the presented case the patient had no history of previous surgery and presented with a persistent intussusception leading to small bowel obstruction.

The current case clearly demonstrates that intussusception should be considered in the differential diagnosis of an adult patient with CD presenting with signs of small bowel obstruction. Hence it is critical to keep intussusception in mind and perform abdominal CT, which is considered as a useful tool for making an accurate diagnosis in this group of patients.

It is stated that identifying the presence or absence of a lead point when dealing with intussusception is crucial. Thus, the former is a serious condition likely to persist and require surgery, while the latter is more likely to resolve spontaneously [9]. When a mass is demonstrated at CT separately from edematous bowel, it can be regarded as a reliable predictor of an intussusception with a lead point and surgery should be recommended. As in the presented case, use of MDCT with multiplanar reformations can be helpful to characterize an intussusception, determine an underlying lead point, and demonstrate evidence of obstruction.

CD can affect any part of the gastrointestinal tract. Appendix is involved in approximately 25% of patients with CD of the terminal ileum and in more than 50% of patients with colonic CD [2]. Inappreciable attention has been given to the CT appearance of the appendix in patients with this condition. Increased appendiceal wall enhancement is more frequently observed in patients with active disease by comparison with patients who have inactive CD [10]. In this case, appendiceal involvement which was shown to be an activity sign in CD was clearly demonstrated. Another sign of activity, the “comb” sign, representing engorgement of the vasa recta was also shown.

This case illustrates an unusual cause of small bowel obstruction in an adult patient with CD. It also demonstrates MDCT findings of appendiceal involvement and signs of active disease.

## References

[1] Gatta G., Di Grezia G., Di Mizio V. (2012). Crohn's disease imaging: a review. *Gastroenterology Research and Practice*.

[2] Shaoul R., Rimar Y., Toubi A., Mogilner J., Polak R., Jaffe M. (2005). Crohn's disease and recurrent appendicitis: a case report. *World Journal of Gastroenterology*.

[3] Shah A., Roberts J., Lipsky H., Twersky Y., Hirth M., Chawla K. (1995). Enteroenteric intussusception: an unusual presentation of Crohn's disease in an adult patient. *The American Journal of Gastroenterology*.

[4] Shaheen K., Eisa N., Alraiyes A. H., Alraies M. C., Merugu S. (2013). Telescoping intestine in an adult. *Case Reports in Medicine*.

[5] Baleato-González S., Vilanova J. C., García-Figueiras R., Juez I. B., de Alegría A. M. (2012). Intussusception in adults: what radiologists should know. *Emergency Radiology*.

[6] Barussaud M., Regenet N., Briennon X. (2006). Clinical spectrum and surgical approach of adult intussusceptions: a multicentric study. *International Journal of Colorectal Disease*.

[7] Greenstein A. J., Wertkin M., Doughlin G., Sicular A. (1979). Enteroenteric intussusception in Crohn's disease. *The Mount Sinai Journal of Medicine*.

[8] Knowles M. C., Fishman E. K., Kuhlman J. E., Bayless T. M. (1989). Transient intussusception in Crohn disease: CT evaluation. *Radiology*.

[9] Tresoldi S., Kim Y. H., Blake M. A. (2008). Adult intestinal intussusception: can abdominal MDCT distinguish an intussusception caused by a lead point?. *Abdominal Imaging*.

[10] Soyer P., Boudiaf M., Dray X. (2010). Crohn's disease: multi-detector row CT-enteroclysis appearance of the appendix. *Abdominal Imaging*.

